# Insufflation pressure above 25 mm Hg confers no additional benefit over lower pressure insufflation during posterior retroperitoneoscopic adrenalectomy: a retrospective multi-centre propensity score-matched analysis

**DOI:** 10.1007/s00464-020-07463-1

**Published:** 2020-02-24

**Authors:** Franck Billmann, Oliver Strobel, Adrian Billeter, Oliver Thomusch, Tobias Keck, Ewan Andrew Langan, Aylin Pfeiffer, Felix Nickel, Beat Peter Müller-Stich

**Affiliations:** 1 Department of Surgery, University Hospital of Heidelberg, Im Neuenheimer Feld 110, 69120 Heidelberg, Germany; 2 Department of Surgery, University Hospital of Freiburg Im Breisgau, Hugstetter Strasse 55, 79106 Freiburg im Breisgau, Germany; 3 Department of Surgery, University Hospital Schleswig Holstein, Campus Lübeck, Ratzeburger Allee 160, 23538 Lübeck, Germany; 4 Department of Dermatology, University Hospital Schleswig Holstein, Campus Lübeck, Ratzeburger Allee 160, 23538 Lübeck, Germany; 5 Department of Dermatological Science, University of Manchester, Manchester, UK

**Keywords:** Adrenalectomy, Retroperitoneal space, Minimally invasive surgical procedures, Surgery

## Abstract

**Background:**

Insufflation pressures of or in excess of 25 mm Hg CO_2_ are routinely used during posterior retroperitoneoscopic adrenalectomy (PRA) in most centres. A critical analysis of the surgical literature provides limited evidence to support this strategy.

**Objective:**

To determine whether high pressure (≥ 25 mm Hg) compared with lower pressure (< 25 mm Hg) retroperitoneoscopy reduces operating time and complications.

**Methods:**

A multi-centre retrospective cohort study was performed using data collected over a period of almost one decade (1st November 2008 until 1st February 2018) from surgical centres in Germany. A total of 1032 patients with benign adrenal tumours were identified. We compared patients undergoing PRA with insufflation pressures of < 25 mm Hg (G20 group) versus ≥ 25 mm Hg (G25 group). A propensity score matching analysis was performed using BMI, tumour size and surgeon's experience as independent variables. The main outcomes were (1) the incidence of perioperative complications and (2) the length of operating time.

**Results:**

The baseline patient characteristics were similar in both groups, with the exception of tumour size, BMI and surgeon's experience in PRA. After propensity score matching, perioperative outcomes, especially perioperative complications (3.7% vs. 5.5% in G20 and G25, respectively; *p* = 0.335) and operation duration (47 min vs. 45 min in G20 and G25, respectively; *p* = 0.673), did not significantly differ between the groups.

**Conclusion:**

Neither patient safety nor operative success was compromised when PRA was performed with insufflation pressures below 25 mm Hg. Prospective studies are required to determine whether an optimal insufflation pressure exists that maximizes patient safety and minimizes the risks of post-surgical complications. Nevertheless, our results call for a careful re-evaluation of the routine use of high insufflation pressures during PRA. In the absence of prospective data, commencing PRA with lower insufflation pressures, with the option of increasing insufflation pressures to counter intraoperative bleeding or exposition difficulties, may represent a reasonable strategy.

Minimally invasive surgery is considered to be the gold standard for the surgical removal of benign functioning and non-functioning adrenal tumours [1–3]. Since its first description [4], minimally invasive adrenal surgery has evolved into two main access techniques: (1) the transperitoneal approach (remote access to the retroperitoneal space through the peritoneal cavity), and (2) the retroperitoneoscopic approach (direct access to the retroperitoneal space through a dorsal or dorsolateral approach). A growing number of endocrine surgical units have adopted posterior retroperitoneoscopic adrenalectomy (PRA) as the first-line approach for small to medium sized benign adrenal tumours [5]. When PRAs were first performed surgeons applied CO_2_ insufflation pressures comparable to those used in the transperitoneal route (12 to 20 mm Hg) [6]. In fact, insufflation pressures of up to 30 mm Hg have subsequently been used based on expert opinion that "the liberal use of" increased insufflation pressures was "one of the essential technologic breakthroughs in PRA (…) to allow the creation of a sufficiently wide space", and would guarantee “a dry operating field, caused by compression of small venous vessels”, and even allow suturing of tears on major vessels such as inferior vena cava [7, 8]. Currently, high retroperitoneal insufflation pressures are used routinely, with nearly all centres using pressures equal to or exceeding 25 mm Hg [6, 7, 8, 9].

Yet, a critical analysis of the literature provides limited evidence to support the use of high insufflation pressures, especially when the disadvantages (hemodynamic, pulmonary, and acid–base metabolic effects) are considered [7, 10]. Although high pressures increase stroke volume, cardiac output, and mean arterial pressure [11, 12], the principal risks include intraoperative hypercapnia and metabolic acidosis [5, 10, 11, 13].

Furthermore, clear evidence that high pressures improve the operative field (consequently reducing operation time) and reduce intra-/postoperative complications is limited.

Recently, the randomized controlled trial by Frazer et al. [14] reported that high retroperitoneoscopic pressures are linked with greater partial pressure of arterial CO_2_ and end-tidal CO_2_ and reduced pH; an effect which was seen 30 min after starting the procedure. Unfortunately, the authors did not investigate whether these effects translated into adverse clinical consequences postoperatively. This is at least conceivable given that operating time for PRA usually exceeds 30 min. Therefore, we hypothesized that high retroperitoneoscopic insufflation pressures may negatively impact operation variables and the postoperative course.

In a retrospective analysis of prospectively collected data from three university surgical centres in Germany, we aimed:

- first to describe a representative series of retroperitoneoscopic procedures performed to treat benign adrenal tumours;
- second to compare patient characteristics and perioperative outcomes in the following groups of patients: (a) high-pressure retroperitoneoscopy (≥ 25 mm Hg; group G25) vs. (b) lower pressure retroperitoneoscopy (< 25 mm Hg; group G20);
- third to determine whether high-pressure (in comparison to low-pressure) retroperitoneoscopy may be linked to significant changes in perioperative outcomes (e.g. hypercapnia);
- fourth to clarify whether insufflation pressure during retroperitoneoscopy influences operation time and reduces the risk of intra- and/or postoperative bleeding.

## Materials and methods

### Study design and setting

This was a multi-centre retrospective cohort study. Our key aim was to compare perioperative outcomes in patients who underwent PRA for benign adrenal tumours (e.g. primary aldosteronism, pheochromocytoma, incidentaloma) using low (< 25 mm Hg; G20 group) versus high (≥ 25 mm Hg; G25 group) CO_2_ insufflation pressures. Retroperitoneal insufflation pressures were defined and applied at the beginning of each procedure, based on surgeon's preference for a given indication or a given patient. The only indication for an increase in retroperitoneal pressure would have been a major intraoperative bleeding. The data of these patients were collected and analysed in an intention to treat analysis.

The study was initiated in 2018, after obtaining an institutional ethics committee approval (ethic approval # S078/2018). The three study centres were located in Germany (Freiburg, Heidelberg, Lübeck) and all have certified and extensive experience in both endocrine and minimally invasive surgery (all centres are certified by the German Board of general and visceral surgery as reference centres for minimally invasive and endocrine surgery) [15]. All centres have access to diagnostic, biochemical and imaging facilities relevant to adrenal diseases and routinely carried out minimally invasive adrenal surgery, with particular expertise in retroperitoneoscopy. Surgical experience in retroperitoneoscopy of the attending surgeons who performed PRA was investigated and used in the propensity matching; 42 procedures being required to fulfil the training requirements to perform this surgery [16].We classified surgeons into two groups (< 42 vs. ≥ 42 procedures performed) at the beginning of observation period or when they were included in the centre's team. There is now robust evidence showing that, with expertise in abdominal surgery and more than 500 minimally invasive surgery (MIS) procedures done, only the experience in PRA can be relevant for the specific outcome investigated in our series [16, 17]. Therefore, as all participating surgeons have performed more than 500 MIS procedures and had an experience of at least 10 years in abdominal surgery, we divided them according to the number of PRAs performed (< 42 vs. ≥ 42 procedures) and matched only in this respect. We collected data from patients, procedures and follow-up between 1st November 2008 and 1st February 2018.

### Cohort and follow-up

Patients included in our study were diagnosed with benign adrenal tumours (e.g. primary aldosteronism, pheochromocytoma, incidentaloma) and underwent PRA. Documented insufflation pressure used to create the retroperitoneoscopic space was mandatory for inclusion in the study. All centres defined a maximum tumour size for retroperitoneoscopic surgery of 6 cm. Follow-up for each patient began on the day of operation. Patients were excluded if (i) they did not complete a standard surgical follow-up of at least 30 days, (ii) they were diagnosed with a malignant adrenal tumour, (iii) there was insufficient documentation of the insufflation pressure which was used.

### Outcomes, study size and bias

The primary outcomes were: (1) any peri- and postoperative complications and (2) the operation duration. Surgical site infections, wound disruptions, seromas, postoperative bleeding, temporary or definitive hypocortisolism and recurrence were considered surgical complications. Conversion to transabdominal laparoscopy or open surgery were considered as secondary outcomes. Furthermore, we recorded the incidence of procedural interruptions requested by anaesthetists to correct hypercapnia. Pulmonary complications (pneumonia, reintubation, or mechanical ventilation), renal conditions (renal insufficiency or acute renal failure), stroke, cardiovascular conditions (cardiac arrest or acute myocardial infarction), thromboembolic conditions (pulmonary embolism or deep venous thrombosis), and infectious conditions (sepsis, septic shock, or urinary tract infections) were considered medical complications.

We gathered demographic, clinical, and perioperative (including pathological and biochemical) data for all patients included in this study. Data on operation duration, blood loss and perioperative complications (using Clavien–Dindo classification [18] and the type of complication) were recorded. Data was also collected on whether surgery was cortex-sparing or not.

A sample size calculation was performed before initiating the study. Given that the study design was based on two independent study groups (retroperitoneal Pressures of ≥ 25 mm Hg vs < 25 mm Hg), and taking account of the primary endpoints and operation duration, (type I/II error rate: alpha = 0.05 and beta = 0.2, power of 80%) a minimum sample size of 103 patients for each group (total study group = 206) was necessary. In order to address bias linked with the retrospective observational design of our study (e.g. differences between groups for tumour size, BMI, surgeon's experience), we performed a propensity score-matched analysis of collected data (see below).

### Statistical analysis

All analyses were done using STATA 15 software (StataCorp, 4905 Lakeway Drive, College Station, Texas 77,845 USA). We summarized continuous variables as mean (Standard Deviation, SD) and median (InterQuartile Range, IQR). Categorial variables were registered as *n* (%). We did statistical comparisons of quantitative variables with Student's *t* test or Mann–Whitney test. For categorial variables, we used the Pearson's *χ*^2^ test or Fisher's exact test. All statistical tests were two-sided, and *p* values of less than 0.05 were considered statistically significant.

A propensity score-matched analysis was performed to minimize selection bias. Propensity scores were calculated using logistic regression. Retroperitoneal insufflation pressure (< 25 vs. ≥ 25 mm Hg) was entered in the regression model as a dependent variable and Age, Sex, BMI, Tumour Size, ASA score, surgeon's experience (< 42 or ≥ 42 procedures) as independent covariates. The cases were matched for their propensity scores using a matching ratio of 1:1, nearest neighbour matching protocol, with a caliper of 0.2. Cases were not reusable after matching.

## Results

### Participant characteristics

**Fig. 1:**
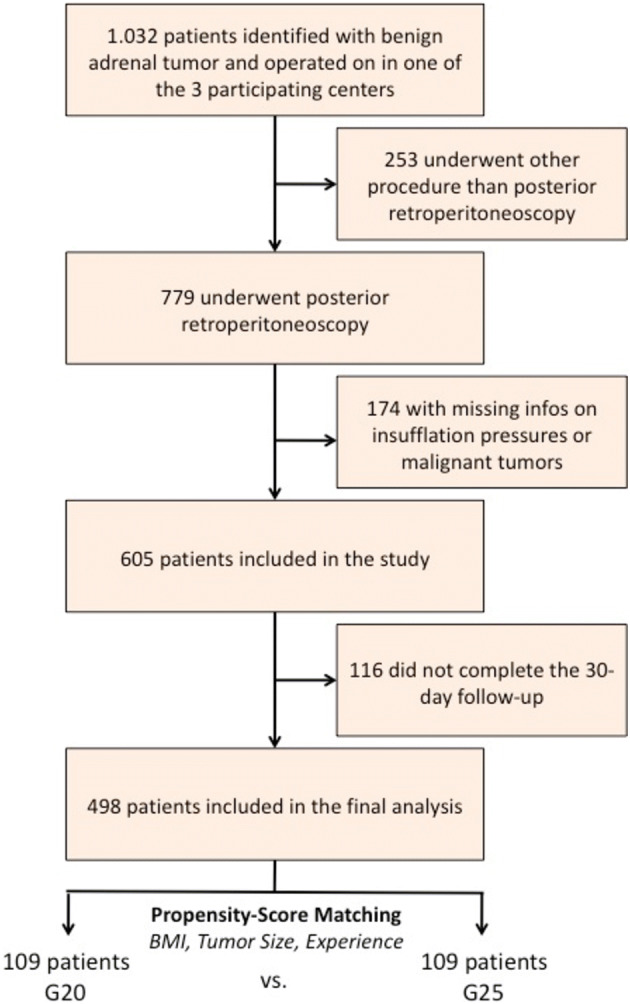
Study flow diagram

In the 3 participating academic medical centres in Germany, a total of 1.032 patients who underwent surgical treatment for adrenal tumours were identified. 253 patients underwent surgical procedures other than unilateral PRA and were therefore excluded from further analysis. In 174 of the remaining 779 patients there was insufficient data on the intraoperative insufflation pressure used to create a pneumo-retroperitoneum. These cases were also excluded from further analysis. 116 patients did not complete the required 30-day follow-up or had a malignant tumour diagnosed (study flow diagram displayed in Fig. 1). The final study cohort consisted of 489 patients with benign adrenal tumours. In terms of demographics, 199 patients (40.7%) were male and 290 (59.3%) were female. Median age at final follow-up was 49 years (IQR 42–56; range 26–80). The patients included in the final analysis had a median follow-up of 24 months (IQR 16–31).

Among included patients, the two defined study groups were composed as follow: 188 (38.4%) had ≥ 25 mm Hg retroperitoneal CO_2_ insufflation pressure (G25) and 301 (61.6%) had < 25 mm Hg pressure (G20). Both groups were statistically comparable for patient characteristics except for tumour size, surgeon's experience and BMI (Table 1). After propensity score matching, the two study group were composed by 109 individual each.

**Table 1:** Patient's characteristics before propensity score matching

Variables	All patients			Propensity-matched patients		
	Retroperitoneal insufflation pressure		*p*-value	Retroperitoneal insufflation pressure		*p*-value
	G20 (*n* = 301)	G25 (*n* = 188)		G20 (*n* = 109)	G25 (*n* = 109)	
BMI, kg/m^2^ (median, IQR)	25.0 (22.3–27.2)	23.6 (21.8–24.0)	**0.048**^**a**^	25.0 (23.2–26.9)	24.9 (23.1–25.3)	0.480^a^
Age, years (median, IQR)	49 (42–56)	48 (42–58)	0.824^a^	49 (43–55)	49 (43–57)	0.852^a^
Sex (*n*, %)			1.00^b^			1.00^b^
Male	123 (40.9%)	76 (40.4%)		44 (40.4%)	44 (40.4%)	
Female	178 (59.1%)	112 (59.6%)		65 (59.6%)	65 (59.6%)	
ASA score (*n*, %)			0.208^c^			0.422 ^c^
ASA 1	136 (45.2%)	95 (50.5%)		50 (45.9%)	54 (49.6%)	
ASA 2	154 (51.2%)	79 (42.0%)		56 (51.4%)	53 (48.6%)	
ASA 3	11 (3.6%)	14 (7.5%)		3 (2.7%)	2 (1.8%)	
ASA 4	0 (0.00%)	0 (0.00%)				
Tumour laterality right (*n*, %)	140 (46.5%)	95 (50.5%)	0.331^b^	50 (45.9%)	55 (50.4%)	0.299^b^
Tumour size, mm (median, IQR)	2.1 (1.5–2.7)	2.3 (1.9–2.9)	**0.005**^**a**^	2.3 (2.0–2.9)	2.3 (1.9–2.9)	0.435^a^
Operator’s experience pe42 PRA (*n*, %)	71 (23.6%)	20 (10.6%)	**0.031**^**b**^	18 (16.5%)	16 (14.7%)	0.541^b^

^a^Mann–Whitney test

^b^Pearson's *χ*^2^ test

^c^Fisher's exact test

*p* < 0.05 were considered statistically significant in bold

### Perioperative characteristics, mortality and morbidity

Perioperative characteristics, mortality and morbidity were analysed after propensity score matching of the included patients. No deaths were observed in the perioperative period and during the 30-day follow-up. The frequency of cortex-sparing surgery was not significantly different between both groups. Perioperative characteristics are summarized in Table 2. Complications were classified according to the Clavien–Dindo classification [18]. Both patient groups were comparable for all investigated perioperative characteristics. In the present series, and after propensity score matching, no conversion to open or laparoscopic surgery was documented in either group.

**Table 2:** Perioperative characteristics after propensity score matching

Variables	Retroperitoneal insufflation pressure		*p*-value
	G20 (*n* = 109)	G25 (*n* = 109)	
OP duration, min (median, IQR)	47 (42–51)	45 (42–50)	0.673^a^
Estimated blood loss, mL (median, IQR)	5 (0–10)	5 (5–15)	0.289^a^
Hospital stay, days (median, IQR)	4 (3–4)	4 (3–4)	0.954^a^
Cortex-sparing surgery (*n*, %)	27 (24.8%)	23 (21.1%)	0.249^b^
Complications (*n*, %)	4 (3.7%)	6 (5.5%)	0.335^b^
Bleeding	1 (0.9%)	0 (0.0%)	
Wound complication (including seroma, wound disruption or infection)	2 (1.8%)	3 (2.8%)	
Definitive hypocortisolism	0 (0.0%)	0 (0.0%)	
Other (including medical complications)	1 (0.9%)	3 (2.8%)	
Intraoperative procedure interruption due to hypercapnia	1 (0.9%)	3 (2.8%)	0.455^c^
Clavien–Dindo [17]			0.496^b^
Grade 1	0 (0.0%)	1 (0.9%)	
Grade 2	3 (2.8%)	4 (3.7%)	
Grade 3A	1 (0.9%)	1 (0.9%)	
Grade 3B	0 (0.00%)	0 (0.00%)	
Grade 4A	0 (0.00%)	0 (0.00%)	
Grade 4B	0 (0.00%)	0 (0.00%)	
Grade 5	0 (0.00%)	0 (0.00%)	
Conversion (*n*, %)			1.0^b^
To laparoscopy	0 (0.0%)	0 (0.0%)	
To open surgery	0 (0.0%)	0 (0.0%)	

^a^Mann–Whitney test

^b^Pearson's *χ*^2^ test

^c^Fisher's exact test

Overall, 10 complications (4.6%) were observed. Perioperative complications were comparable in both groups.

## Discussion

Our study convincingly demonstrates that perioperative complications (especially bleeding) and mortality, as well as procedure duration are independent of the insufflation pressure used when comparing high (≥ 25 mm Hg) with low pressures (< 25 mm Hg).

### Operation duration

In our cohort (G20 and G25 together), the median overall operation duration of 47 min, is comparable with other series in the literature where operation time ranges from 51 to 157 min [8, 9, 19–21]. Operative time was not significantly different between the groups (median of 47 min vs. 45 min in G20 vs. G25, respectively; *p* = 0.673). This result stands in contradiction to the results of the majority of publications reporting the association between increased insufflation pressure and faster procedures [7, 22], where authors concluded higher insufflation pressures to be an "essential technologic breakthroughs in PRA" to allow " the creation of a sufficiently wide space" and the guarantee of “a dry operating field” [7, 19, 22]. Propensity score matching matched both groups (G20 and G25) for possible confounding factors. Thus, our results may suggest that pressures of or higher than 25 mm Hg do not warrant an exposure advantage (and thus reduce operating time) as proposed by other minimally invasive surgeons specialized in adrenal surgery [7, 8]. Based on these expert statements, most studies dealing with PRA insufflation pressure did not investigate the usefulness of high pressures (≥ 25 mm Hg) in a statistically robust manner. The current literature is also likely to be subject to selection bias, with pressure groups analysed which were not statistically comparable. Our investigation overcomes this problem using a propensity score matching (G20 vs. G25), making the present investigation particularly valuable. When specifically asked, surgeons at participating centres did not subjectively experience any change of operative field exposure in the G25 group when compared to G20 (unfortunately our study was not designed to specifically assess this effect).

## Postoperative complications

Overall 10 complications (4.6%) were observed in our matched series, one patient having potentially more than one complication. The rate of complications is comparable to other series in the published literature. The groups did not significantly differ in terms of perioperative complications, with the exception of procedure interruption due to intraoperative hypercapnia, which is not a component of the Clavien–Dindo classification. Thus, PRA for benign tumours of the adrenal gland performed under low pressure (< 25 mm Hg) had a safety profile similar to that carried out under high insufflation pressure (≥ 25 mm Hg and above). Subcutaneous emphysema was not systematically recorded in the present series. Thus, we cannot make any comparison between low- versus high-pressure retroperitoneal insufflation. As this emphysema usually resolves within hours of the procedure, we might suppose it does not have a major impact on patient wellbeing or satisfaction.

Another intraoperative complication was an unintended pneumoperitoneum during the retroperitoneal procedure. Although this complication was not systematically registered, procedure records did not describe major problems in the completion of the retroperitoneal dissection. Due to the lack of uniformity in retrospective evaluation of its consequences among groups, we were not able to quantify any difference between high- versus low-pressure insufflation with regard to this specific complication. Moreover, we did not register any conversion to open surgery in this matched series.

Whereas the effects of pneumoperitoneum on ventilatory and metabolic parameters have been well documented and are now well appreciated, the physiological effects of the CO_2_ retropneumoperitoneum (e.g. ventilatory and metabolic effects) have seldom been systematically investigated [11, 23, 24]. Furthermore, most studies were performed at low CO_2_ insufflation pressures of 12 to 16 mm Hg [10], not comparable with actual operative settings (use of pressures above 20–25 mm Hg)*.* Comparison between CO_2_ insufflation pressures of 15 and 20 mm Hg showed a statistical but not clinically relevant difference in PaCO_2_ [11]. As most endocrine centres use retroperitoneal pressures of more than 25 mm Hg, these results have to be interpreted with caution [5].

Although postoperative complications did not differ among groups in our analysis, the operative procedure had to be temporarily interrupted due to hypercapnia in 2.8% of cases in G25 and 0.9% of cases in G20 (*p* = 0.455). As the present study was not designed to investigate postoperative consequences of intraoperative hypercapnia, we are not able to definitively draw any conclusions about this effect, which warrants examination in future studies. The prospective randomized study by Frazer et al. demonstrated a direct relationship between retroperitoneal CO_2_ insufflation pressure, hypercapnia and acidosis [14]. The authors found high pressures (25 mm Hg in comparison to low pressure of 20 mm Hg) was linked to greater mean partial pressure of arterial CO_2_ (64 vs. 50 mm Hg, *p* = 0.003), end-tidal CO_2_ (54 vs. 45 mm Hg, *p* = 0.008) and a lesser pH (7.21 vs. 7.29, *p* = 0.0005), results which contradict previous investigations [11]. This effect was significant after 30 min of operative time [14]. As most PRAs take longer than 40 min, the consequences of intraoperative hypercapnia have to be carefully considered, and the surgeon should expect significant ventilatory and metabolic consequences when using high CO_2_ insufflation pressures. However, demonstration of this intraoperative phenomenon in large clinical case series is still lacking. In order to detect significant clinical differences in patient outcome, recovery and complication when using high CO2 pressures and in order to contravene a one-sided assessment of the problem, large sample size is needed. Finally, we concur with Frazer's recommended strategy of start the PRA procedure with lower CO_2_ insufflation pressures in order to decrease intraoperative PaCO_2_ and acidosis, and to increase only if the surgeon experiences bleeding or exposition difficulties. Patients with greater comorbidities may require an even stricter control of PaCO_2_ and pH [5]. In this specific group of patients, differences in intraoperative hypercapnia levels may have greater impact and a careful and active monitoring of acid–base balance and PaCO_2_ is therefore essential, as an important part of this procedure [25]. The cardiovascular consequences of hypercapnia may be of particular importance in PRA, when patients are undergoing operation for a pheochromocytoma [5]. Furthermore, as the prevalence of adrenal incidentaloma increases with patient age, the greater prevalence of background cardiovascular disease in this ageing population must also be considered when performing PRA with greater CO_2_ insufflation pressures and the associated hypercapnic acidosis. Although we did not design our study to detect any clinically apparent effect of hypercapnia on physiologic outcomes, it is worth noting that clinically important outcomes of these changes may certainly occur.

### Intraoperative blood loss

In our series, estimated intraoperative blood loss (mean = 10.4 mL) was comparable with previous published series [22, 26–29]. There was no significant difference between the groups (median of 5 mL for G20 and G25). Thus, a retroperitoneoscopic pressure of < 25 mm Hg seems to be sufficient to maintain a dry operative field. As we did not specifically investigate insufflation pressures increments, we cannot make any conclusions regarding low pressures (especially those around 15–20 mm Hg). However, it is important to note that high pressures (up to 25 or 30 mm Hg) may still be necessary in order to handle active bleeding (e.g. bleeding of the vena cava inferior).

### Postoperative hospital stay

Consistent with the length of hospital stay in published case series [26, 28, 30], our patients mean postoperative hospital stay was 4.06 days in comparison to 1.6–4.5 days in the literature. In our series, there was no significant difference among groups (G20 vs. G25). In analogy with blood loss, this result does not support higher retroperitoneal insufflation pressures to be essential or mandatory for faster recovery. However, the present study was not designed to specifically investigate this effect.

## Bias and Limitations

The main limitation is that this study was a retrospective analysis. In addition,

retroperitoneal insufflation pressures were documented at the beginning of each procedure and not subsequently modified (see methods section). Therefore, we do not consider this factor to be a source of bias or major limitation in the present study. However, patients were not randomly assigned to each of the insufflation pressure groups; thus, there is a relative weakness in the present study design, which may have impacted on the results.

We investigated performance bias by stratifying patients by surgeon and/or surgical centre. As we could not exclude performance bias, we entered surgeon's PRA experience as independent variable in our propensity score matching analysis. Another potential limitation of our study was the statistically significant difference in the tumour size and BMI between both studied patient cohorts. Thus, we treated both variables as independent variables in our propensity score matching analysis.

In addition, the variable follow-up periods represent a minor limitation of our study in terms of procedure safety. For example, patients operated on with a ≥ 25 mm Hg pressure had a median of 26 months of follow-up, while those who had operations with a < 25 mm Hg pressure had only a median of 19 months of follow-up (*p* < 0.001). To assess the severity of this bias, we ran sensitivity analyses that included only patients who strictly met a 2-year follow-up period. We found that there was minimal bias in our analysis.

It is important to notice that pressures between 20 and 25 mm Hg can still be considered to be a relatively high pressure (most PRA being performed with a pressure around 20 mm Hg) when compared with normal intraperitoneal laparoscopy pressure (around 16 mm Hg). An insufflation pressure > 25 mm Hg should be considered even more to be very high. Thus we suggest adopting a strategy based on a moderate pressure elevation starting with 20 mm Hg or less (e.g. 18 mm Hg). That strategy may be sufficient to achieve better exposition without exposing the patient to the risks of pressures > 25 mm Hg. Due to its design, our study is not able to determine whether normal laparoscopy pressures (around 16 mm Hg) are sufficient to perform the PRA procedure safely.

Finally further limitations to our study include (i) exclusion of patients who did not fulfil the 30-day follow-up and (ii) the restriction of our analysis to patients with benign tumours. These factors may both have led to a degree of selection bias. Further studies are required to specifically investigate the observed effects in other clinical settings (e.g. malignant tumours).

## Conclusion

Our study shows that insufflation pressures below 25 mm Hg do not compromise patient safety or operative success in PRA. Moreover, moderate initial pressure (20 mm Hg or less), followed by increased pressures where required, may be sufficient to achieve better exposition and to overcome intraoperative bleeding. Taken together, our results call for the careful re-evaluation of the need for routine high insufflation pressures; a practice based on sparse clinical evidence. Prospective investigations are required to definitely determine whether an optimal insufflation pressure exists which allows both safe completion of the procedure and improved patient outcomes.

## Abbreviations

PRA: Posterior retroperitoneoscopic adrenalectomy
mm Hg: Used for mm Hg CO_2_
IQR: InterQuartile range
SD: Standard deviation
ASA: American Society of Anaesthesiologists
PaCO2: Partial pressure of carbon dioxide in the arterial blood
